# The Association between Maternal Stress and Childhood Eczema: A Systematic Review

**DOI:** 10.3390/ijerph15030395

**Published:** 2018-02-25

**Authors:** Carmen W. H. Chan, Bernard M. H. Law, Yun-Hong Liu, Alexandra R. B. Ambrocio, Natasha Au, Melody Jiang, Ka Ming Chow

**Affiliations:** 1 The Nethersole School of Nursing, The Chinese University of Hong Kong, Shatin, the New Territories, Hong Kong, China; whchan@cuhk.edu.hk (C.W.H.C.); liuyunhong@link.cuhk.edu.hk (Y.-H.L.); 2 Department of Psychology, New York University, New York, NY 10012, USA; alexandra.ambrocio@nyu.edu; 3 Department of Psychology, The University of British Columbia, Vancouver, BC V6T 1Z4, Canada; natashaau@alumni.ubc.ca; 4 Department of Interdisciplinary Studies, University of North Carolina at Chapel Hill, Chapel Hill, NC 27599-2200, USA; bmjiang@unc.edubinyumjiang@gmail.com

**Keywords:** childhood eczema, atopic dermatitis, maternal stress

## Abstract

Eczema is a chronic atopic disease that is highly prevalent among children worldwide. Identification of factors that may contribute to childhood eczema is needed in order to develop strategies in its prevention. Over the past decade, accumulating evidence has suggested a potential correlation between the experience of stress by mothers and the risk of eczema development in their child. The present review attempts to provide an overview of the studies that contribute data on this correlation. The literature search was conducted using five databases, resulting in the inclusion of eleven studies in the review. The findings of these studies were summarized narratively. Further, an appraisal of the reporting quality of the included studies was conducted using a twelve-item checklist adapted from the Strengthening the Reporting of Observational Studies in Epidemiology (STROBE) checklist. Overall, the included studies showed that a positive correlation exists between the experience of stress among mothers and eczema risk of their child. The findings highlight the importance of the implementation of stress reduction programs for pregnant women and those in their postpartum period within communities in order to enable these individuals to relieve stress effectively.

## 1. Introduction

Eczema, or atopic dermatitis, is a common chronic disease characterized by the inflammation of the skin. It is estimated to have affected 40 million individuals worldwide [1], and its prevalence is still increasing. Notably, eczema appears more prevalent among children under five years of age, where 20% of children worldwide are suffering from eczema [1], and its prevalence decreases with advancing age [2]. Its clinical features include epidermis dehydration at the affected sites [3], vesicle formation, excoriation and oozing of the reddened, scaly and crusty skin. Owing to these eczema-associated symptoms, eczema patients would normally experience a considerable reduction in their quality of life (QOL), primarily caused by pruritus, sleep disruption and a reduction of psychosocial well-being [1]. Moreover, eczema may impose a considerable economic burden for patients and their families, due to the huge cost incurred by the purchase of medication required for symptom management and skin care [4]. Owing to the high prevalence of eczema among children and the detrimental impact of the disease to patients’ QOL, it is of paramount importance to identify the factors that are causal to childhood eczema. Over the past years, studies had identified a number of factors that are associated with the increased risk of childhood eczema. One of the notable factors identified recently is the experience of stress by mothers, which is generally termed maternal stress.

Stress is normally induced by a stimulus of a stressor which may include traumatic life events, and undesirable home or work environments, which would lead to the activation of the hypothalamic pituitary adrenal (HPA) axis and cortisol production, and potentially increased risk of postpartum depression [5]. Indeed, earlier studies had demonstrated the ability of maternal cortisol to cross the placenta to the fetus [6], where it can exhibit a repertoire of detrimental effects including impaired brain development and low birthweight of the offspring [7], thereby providing evidence for the undesirable effects of stress during pregnancy on the offspring. However, more recent studies also showed that maternal stress may also be linked to childhood eczema risk. Consistent with this, maternal stress was demonstrated to contribute to the deregulation of the immune function in the offspring. This would subsequently lead to inflammation [8], a condition linked to the occurrence of eczema [9]. Further evidence for the relationship between maternal stress and eczema risk in offspring is provided by another study demonstrating that maternal stress would lead to the production of cytokines that would contribute to the development of allergies [10]. In other words, immune dysregulation may serve as a link between stress in mothers and increased risk of their children having eczema. With an increasing number of studies pointing towards the link between these two parameters over the past few years, a systematic review summarizing the findings of these studies on this link would be worthwhile.

Previously, a systematic review on the association between the experience of maternal stress during pregnancy and childhood eczema had been published by Andersson et al. in 2016 [11]. Nevertheless, the authors summarized the findings of the studies on the association between prenatal maternal stress and various types of atopic diseases, rather than eczema alone, in the review. Owing to the heterogeneous nature of the included studies in the review, a firm conclusion on such association was difficult to be drawn. In the review, the authors focused only on prenatal maternal stress. Studies that examine the association between childhood eczema risk and maternal stress at pre-conception and that at postpartum period were not included. A general picture on whether an association exists between maternal stress and childhood eczema risk is therefore lacking. Furthermore, there have also been a number of observational studies examining the relationship between maternal stress and childhood eczema risk published after the publication of the review by Andersson et al., contributing further data on such relationship using samples from various countries. Therefore, another systematic review on this topic is still required. The aim of our review is to provide an overview of the findings of previous studies specifically examining the association between the experience of maternal stress at pre-conception, prenatal and postpartum stages and the occurrence of eczema or atopic dermatitis among children. Specifically, we sought to find out whether the experience of a higher level of stress by mothers would lead to increased risk of their children developing eczema.

## 2. Methods

### 2.1. Search Strategy

A comprehensive literature search was conducted in September 2017. Five databases were used during the literature search, including PubMed, OVID MEDLINE (since 1946), EMBASE, PsycINFO (since 1806) and CINAHL. The combination of keywords used for the literature search is shown as follows: “eczema” or “atopic dermatitis” and “maternal stress” or “prenatal stress” or “psychological stress” or “psychosocial stress” or “anxiety” or “prenatal depression” or “postpartum depression” or “postpartum blue”.

### 2.2. Eligibility Criteria

Included studies in the review are required to be original research articles that report observational cohort studies on the association between the following two parameters: the experience of any forms of stressors by mothers which leads to maternal stress and the odds of their infants developing eczema or atopic dermatitis during childhood. Studies that report cross-sectional, prospective and retrospective cohort studies, including those involving secondary data analysis, were included. Studies that do not report the association mentioned above were excluded. Moreover, studies that are not published in English were also excluded. All relevant and eligible studies that were published before September 2017 were included in this review.

### 2.3. Data Extraction and Synthesis

Using the search strategy described above, one author first selected the relevant and eligible studies to be included in the review by screening the titles and abstracts of the extracted studies. The inclusion of the extracted studies was then verified by four other authors. The full-text articles of the abstracts that match the eligibility criteria were then examined to confirm the eligibility of these articles to be included in the review.

Data extraction was performed by one author after the finalization of the inclusion of studies for the review. Verification of the extracted data was then performed by four other authors in order to ensure the accuracy of the extracted data to be presented in the review. Extracted data include study settings, study design, cohort size, the stress factor investigated the confounding factors involved in the included studies, and the odds ratios (OR) or relative risk (RR) of the aforementioned association. A total of three items of disagreements occurred after the verification of the extracted data by the authors, and they were resolved by discussion between the authors in order to reach a consensus.

Owing to the heterogeneity of the stress factors involved, a meta-analysis on the extracted data was not feasible. Therefore, the findings of the included studies were presented in a narrative and tabular form. If applicable, both the adjusted and unadjusted OR or RR reported in the included studies were presented. Further, the significance of the aforementioned association reported in the studies was presented in the form of *p* values.

### 2.4. Quality Assessment of Included Studies

The reporting quality of the observational studies included in the review was assessed using twelve selected items adopted from the Strengthening the Reporting of Observational Studies in Epidemiology (STROBE) checklist for cohort studies [12]. This assessment primarily assessed the comprehensiveness of the reporting of the methodologies used and findings generated in the included studies. This serves to identify the included studies that may have to be excluded due to the lack of the completeness in the reporting of the undertaken research, and thus the lack of reliability of the reported data. The reporting quality of the studies was first assessed by one author, and the assessment results were then independently verified by four other authors. Percentage agreement in the ratings of the studies’ reporting quality among the five authors was 93%, and disagreements in the ratings between the authors were resolved through discussion to reach a consensus. Selected items in the assessment include whether the included studies have provided an adequate description of the following: (1) the basic characteristics of the studies, including study design and settings, rationale of sample size used and eligibility criteria used in subject recruitment; (2) the methodological details, including the variables, sources of measures used, statistical methods used and how the authors address biases; (3) the outcomes of the studies, including the number of participants involved at each stage of the study, the demographic and clinical characteristics of participants, the data obtained from the measures utilized in the studies, and the unadjusted and/or adjusted estimates obtained from these data. One mark was awarded for each item in the checklist if such item was achieved in the studies. Any items that were not achieved by the studies were given a zero score. The number of achieved items for each included study was then expressed as the total reporting quality score for the study, with the highest score being twelve. Included studies awarded with a total reporting quality score of six or less were regarded as poor quality and were excluded from the review.

## 3. Results

### 3.1. Search Results

**Figure 1:**
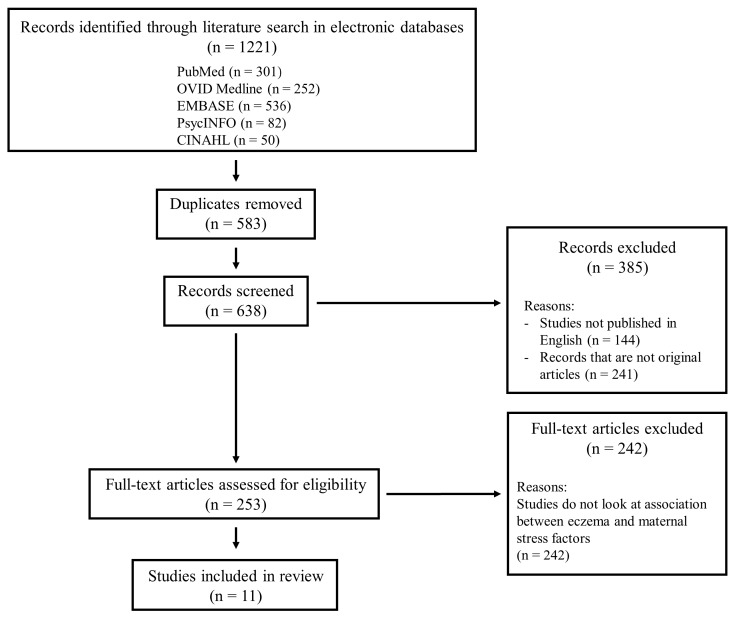
The PRISMA flow diagram.

In total, 1221 publications were identified through the literature search using the search strategy described above. Of these 1221 publications, 583 were duplicates and were therefore excluded. A further 385 publications were removed as they are either not original articles or those that are not published in English. The abstracts of the remaining 253 publications were then screened to extract the relevant articles that report observational cohort studies examining the association between eczema or atopic dermatitis and maternal stress. With the further exclusion of 242 articles which do not meet these criteria, 11 studies were included in the review. The full-text of these 11 studies were then examined to ensure that they meet the eligibility criteria of the review. The PRISMA flow diagram showing the different stages of the study selection process is presented in Figure 1.

### 3.2. Quality of the Included Studies

The ratings of the quality of the studies in reporting observational studies are presented in Table 1. The quality of the reporting of observational studies exhibited by the included studies is generally high, with scores ranging from seven to eleven, out of the highest possible score of twelve. All of the included studies have provided an account on the instruments used for measuring the outcomes involved and statistical methodologies used in the method section, and they presented the outcomes that had been adjusted for the identified covariates or confounding factors. Further, all these studies have provided information on the characteristics of the participants involved in the studies and the main results in the form of unadjusted and/or adjusted estimates. Interestingly, however, none of the studies have included a discussion on the rationale on the sample size used in their studies. Moreover, a number of studies that involve the utilization of data taken from datasets obtained from previously published studies were found to omit the reporting of certain required elements as set out in the STROBE checklist. In these cases, the authors only provided a reference for readers to refer to for the details of these previous studies.

Moreover, upon inspection of the methodology of the data collection reported, we found that most of the included studies utilized a self-report approach for data collection. For the methodology in the ascertainment of childhood eczema, all of the included studies primarily utilized data obtained from parental self-report. Likewise, the included studies involve the use of questionnaires to collect data on maternal stress exposure among the study participants, although some of these studies [13,14,15,16,17,18,19] utilized items adopted from validated instruments in their questionnaires. With the use of a self-report approach in data collection, the findings of these studies could therefore be prone to response bias, and they have to be based on the assumption that all participants responded to the questions truthfully. Nevertheless, in assessing the level of maternal stress exposure, Sausenthaler et al. [20] utilized information from the maternity certificates containing records of previous pregnancy complications that were reported by health professionals during medical checkups. Braig et al. [17] also compared the results obtained from the self-report approach and those obtained using objective measurements such as hair cortisol levels. These approaches may help limit the level of response bias in the collected data, and therefore enhances the reliability of the findings.

### 3.3. Characteristics of the Included Studies

The characteristics of the included studies are listed in Table 2. The publication year of these studies ranged from 2009–2017. Three studies were conducted in Asia (one in South Korea [13] and two in Taiwan [14,15]), one in Canada [16], one in Australia [21], and the remaining six studies were all conducted in Europe (one in Denmark [22], two in Germany [17,20], one in Holland [18], one in Italy [23] and one in the United Kingdom [19]). The majority of the included studies (*n* = 8, 73%) are prospective cohort studies, with a longitudinal design [13,14,15,17,18,19,21,22]. The remaining three studies are retrospective cohort studies, with either a longitudinal [20] or cross-sectional design [16,23]. Two of these studies [16,20] involve secondary data analysis of previous studies.

Participants in the included studies are generally previously pregnant women with at least one child. The sample size utilized in these studies ranges between 242 and 32,271 mother-child pairs. The average sample size is 6474 mother-child pairs.

The included studies have involved the investigation of the association between a variety of stress factors experienced by mothers and eczema or atopic dermatitis risk among their child. These stress factors include perceived stress and psychological distress at pre-conception [19], prenatal anxiety and/or depression [13,16,17,18], stress during pregnancy [15,17,20], undesirable life events [21,23], postpartum depression [14] and job strain during pregnancy [22]. To assess the occurrence of eczema or atopic dermatitis, all studies utilized self-report of eczema-associated symptoms in the form of a questionnaire as a means of assessment of eczema. However, some studies primarily assessed the occurrence of childhood eczema based on parentally-reported diagnosis of eczema by a physician [16,18]. In addition, four studies performed such assessment using a combination of self-report of eczema-associated symptoms and physician diagnosis of childhood eczema [14,15,20,21]. Nevertheless, one study [13] involved the use of two cohort studies, which utilized either clinical diagnosis or self-report of symptoms as a means of childhood eczema assessment. 

### 3.4. Reported Association between Eczema and Maternal Stress

A summary of the findings of the included studies on the association between various maternal stress factors and childhood eczema risk is presented in Table 2. The outcome measures for assessing the stress factors and the methodologies utilized for childhood eczema assessment among the included studies do exhibit a considerable degree of heterogeneity. Nevertheless, the findings on the association between the experience of stress among mothers and eczema risk of their children reported by these studies are generally consistent. After adjusting for confounding factors, all the studies found that at least one of the stressors investigated show a positive association with childhood eczema risk, although only some of the studies (*n* = 8, 73%) were able to demonstrate that the association is significant. The reported adjusted ORs/RRs range from 0.6 to 4.2, although most of the studies reported an adjusted ORs that range between 1.0 and 1.5, suggesting that maternal stress factors demonstrate a weakly positive correlation with childhood eczema risk.

#### 3.4.1. Depression

Four studies [13,14,17,18] investigated the association between the occurrence of prenatal or postpartum depression in mothers and eczema risk of their child. Interestingly, inconsistent results were obtained from these studies. While Wang et al. [14] and Chang et al. [13] reported a significantly increased eczema risk among children whose mothers had suffered from postpartum or prenatal depression respectively, the other two studies [17,18] failed to report significant association between the two parameters, even though a positive association was reported. Moreover, using the atopic dermatitis diagnosis that was strictly defined by dermatologists and an assessment of depression with the depression sub-scale of the Hospital Anxiety and Depression Scale, Braig et al. [17] even demonstrated a lowered risk of childhood eczema among children with mothers suffering from depression at pregnancy, although the decrease was not significant (adjusted RR: 0.6, 95% CI: 0.2–2.3). The reported ORs or RRs for this stress factor range from 0.6–1.4.

#### 3.4.2. Anxiety

Four studies [13,16,17,18] examined the correlation between the experience of prenatal anxiety in mothers and eczema risk in their child. Three of these studies [13,16,18] were able to demonstrate a significant positive correlation between these two parameters. Nevertheless, Braig et al. demonstrated that little or no positive correlation exists between the experience of anxiety by mothers during pregnancy and atopic dermatitis risk in their child. The reported ORs or RRs for this stress factor range from 1.1–2.7.

#### 3.4.3. Maternal Stress

Fairly consistent results were obtained from the three studies [15,17,20] that examined the association between maternal stress in mothers and eczema risk in their child. Although Braig et al. [17] reported a lack of association between chronic stress in mothers and the occurrence of atopic dermatitis in their child, Wen et al. [15] and Sausenthaler et al. [20] showed that mothers who experienced stress during their pregnancy would have significantly increased odds of having a child suffering from atopic dermatitis at 2 years old. Nevertheless, Braig et al. [17] showed that when atopic dermatitis diagnosis was based on the exhibition of atopic dermatitis-associated symptoms and not the definition by dermatologists, the increased risk of the child having atopic dermatitis would become borderline significant with the experience of increased chronic stress in mothers. The reported ORs or RRs for this stress factor range from 1.1–2.3.

#### 3.4.4. Adverse Life Events

Two studies, published by Hartwig et al. [21] and de Marco et al. [23], showed that experience of adverse life events by mothers during their pregnancy would lead to increased odds of their child developing eczema during their childhood. Moreover, Hartwig et al. [21] further demonstrated that the experience of these adverse life events at later stages of gestation (18–34 weeks of gestation) by mothers would have a more profound effect in increasing the odds of the child developing eczema, particularly when the child reaches adolescence. The reported ORs for this stress factor range from 1.2–4.2.

#### 3.4.5. Others Examined Stress Factors

Other stress factors that were investigated by the included studies of this review include perceived stress [19], psychological distress [19], prenatal distress [13] and job strain during pregnancy [22]. Apart from psychological distress, all the other three factors appear to show significantly positive correlation with childhood eczema risk. Moreover, perceived stress was found to exhibit more significant positive association with childhood eczema risk when the child grows older [19]. The reported ORs for these four stress factors range from 1.1–1.9.

Notably, three studies [19,20,21] had reported the temporal changes of the ORs at various data collection time points, thereby shedding light on whether the extent of the reported positive association between the experience of various stress factors by mothers and eczema risk of their child would change as the child grows. Interestingly, conflicting data were reported among these three studies. El Heis et al. [19] showed that the perceived stress and the experience of psychological distress among mothers had an increasingly significant positive association with eczema among their infants as they grew from six-month-old to one-year-old. Likewise, Hartwig et al. [21] demonstrated that the odds of 14-year-old children having eczema were considerably higher than six-year-old children if their mothers had experienced an increased number of adverse life events during late gestation. In contrast, an earlier study conducted by Sausenthaler et al. [20] revealed that although maternal stress during pregnancy appeared to be positively associated with childhood eczema risk at all time points of data collection, the significance of this positive correlation was only observed among younger children below the age of two. This observation suggests that maternal stress factors exhibit reduced association with childhood eczema risk as the child grows older, which contradicts with the findings by El Heis et al. and Hartwig et al.

## 4. Discussion

It has previously been well established that maternal stress would exhibit detrimental effects on infants, such as developmental defects and impaired behavioral development [24,25]. However, over the past decade, an increasing number of studies have started addressing the question on whether such detrimental effects may include an increased risk of the development of atopic diseases such as eczema or atopic dermatitis. This review serves to provide an overview of the data from observational studies on whether the experience of stress by mothers at pre-conception, prenatal and postpartum stages is associated with the odds of their child to develop eczema.

### 4.1. Interpretation of the Review Findings

The findings of the present review demonstrate that a variety of stress factors experienced by mothers would generally lead to an increased risk of their child developing eczema, and eight of the included studies showed that such increase was statistically significant. These stress factors include prenatal depression, postpartum depression, prenatal anxiety, maternal stress during pregnancy, prenatal adverse life events, job strain during pregnancy, prenatal distress and perceived stress. Nevertheless, heterogeneity of the findings does exist among the included studies. Notably, contradictory results were reported by studies that investigated the association between depression and childhood eczema risk [13,14,17,18], as described above. Further, it is still unclear how the childhood eczema risk would be altered once the child whose mother has experienced maternal stress grows older, owing to the contradictory results obtained from studies assessing the temporal changes in the extent of the association between maternal stress and childhood eczema risk [19,20,21]. 

Although being speculative, it is possible that the utilization of individuals of various ethnic groups among the studies could have contributed to the discrepancies, as it was cited that individuals in different racial groups, albeit in the same country, appear to exhibit variations in the tendency of allergy development [26]. In the present review, we found that while studies carried out in European countries [17,18] failed to identify an association between depression and childhood eczema risk, those conducted in Asian countries [13,14] were able to demonstrate the association. It is likely that the ethnic disparities in the tendency of eczema development might have affected the degree of the reported association between maternal stress and childhood eczema risk between the included studies involving participants of various ethnic groups.

Nevertheless, we cannot rule out the possibility that the heterogeneity of the methodologies utilized in examining the association mentioned above could also play a part in the discrepancies. First, various included studies have identified different sets of confounding factors that may affect the measured outcomes, and the reported ORs that have been adjusted for were based on these identified sets of confounding factors. As the confounding factors accounted for were different between the studies, this could potentially lead to variations in the statistically-determined ORs among the studies, which in turn leads to variations in the extent of the significance of the positive association determined by the different studies. Second, the methodology used for the ascertainment of eczema was different between the studies. Notably, in the Braig et al. study [17], the authors utilized two strategies in ascertaining whether the child has developed eczema—through the observation for eczema-associated symptoms and the strict definition of eczema. While an almost significant association between maternal chronic stress and childhood atopic dermatitis risk was observed when symptom observation was used as a strategy for atopic dermatitis ascertainment (adjusted RR: 1.5, 95% CI: 1.0–2.3, *p* = 0.05), such association was determined to be insignificant when the strict definition of atopic dermatitis was used (adjusted RR: 1.1, 95% CI: 0.7–1.9). In light of the potential of the utilization of various methodologies for the measurement of outcomes and that of subjects of various ethnic groups among the studies in yielding heterogeneous findings, caution should be exercised in the interpretation of the findings of the review. Nevertheless, the included studies have in general shown that maternal stress appears to show a positive association with childhood eczema risk. To confirm this, further studies need to be conducted, involving subjects with different ethnic groups, to examine the aforementioned association using universal methodologies in assessing the occurrence of eczema and stress measurement.

One further point of note is that the current number of studies that examine the association between the experience of maternal stress factors at the pre-conception stage and the postpartum period and childhood eczema risk is too small. Therefore, although our review points towards a generally increased eczema risk among children whose mothers have experienced maternal stress during their pregnancy, it is difficult to draw a conclusion on whether the experience of stress by mothers at pre-conception or postpartum stages would increase childhood eczema risk. Further studies examining the association between the experience of stress at these two stages and childhood eczema risk are warranted to address this issue.

### 4.2. Implications of the Review Findings

Owing to the high prevalence of childhood eczema worldwide, the effective prevention of the disease among the young children is of paramount importance. With the findings showing that maternal stress would generally exhibit a positive correlation with childhood eczema risk, strategies should be taken to ensure that exposure to stressful conditions by mothers should be kept to a minimum, both during their pregnancy and postpartum period. For example, education of pregnant women on stress reduction should be enhanced. Health talks should be regularly held for pregnant women in communities, disseminating knowledge on the effective strategies to relieve stress and deal with adverse life events, such as regular exercises. The importance of seeking social support and professional help immediately when signs of anxiety and depression appear during pregnancy and postpartum period should also be widely disseminated among pregnant women. Further, as job strain during pregnancy was found to be a factor that increases the risk of childhood eczema, pregnant women should also be encouraged to take the mandatory paid maternity leave during the period of pregnancy. Pregnant women should also refrain themselves from working overtime for higher wages, as long working hours were previously suggested to be causal to anxiety and depression [27]. All these strategies should be effective in preventing pregnant women from exposure to excessive stress, and may therefore reduce the risk of their child from developing eczema during their childhood.

## 5. Limitations

The present review has some limitations that need to be acknowledged. First, as mentioned above, the differences in the ethnic groups of the subjects and the methodology in the ascertainment of childhood eczema among the included studies of this review could have contributed to the heterogeneity of the review findings, resulting in the difficulties in drawing firm conclusions on the association between the experience of maternal stress and childhood eczema risk. Second, all of the included studies utilized a self-report approach, largely in the form of a questionnaire, in the determination of stress exposure among mothers and/or ascertainment of eczema development in their child. The obtained data using this subjective approach of data collection would therefore be prone to recall bias, limiting the reliability of the findings. Future studies should consider the use of objective measurement strategies in the assessment of these outcome measures, as suggested by Andersson et al. [11]. For example, hair cortisol levels could serve as an objective and reliable measure for stress [28]. Moreover, technological advances have also enabled the development of wearable sensors for the monitoring of chronic stress, based on data reflecting the heart rate variability, in an objective manner [29]. In addition, future studies should employ physician-diagnosed eczema as the strategy in the process of ascertainment of eczema development among the participants, instead of mere observation on whether eczema-associated symptoms have appeared, in order to determine more accurately whether the participants have developed eczema. Third, the included studies generally utilized convenience samples for subject recruitment. This may potentially lead to selection bias, where the eligible participants may possess similar characteristics of a particular confounding factor identified in the studies. This in turn leads to limitations in the generalizability of the findings, despite the involvement of a large sample size in the studies. Future studies on assessing the association between maternal stress and childhood eczema risks may therefore consider the utilization of samples recruited from multiple sites, in various parts of the country where the study is conducted, despite its inconvenience of being labor-intensive. 

## 6. Conclusions

Over the past years, an increasing number of studies have addressed the question on whether the experience of stress by mothers during their pregnancy would detrimentally affect their children by increasing their risk in developing eczema, an atopic disease that is highly prevalent among children worldwide. The findings of the included studies in the present review generally point towards a higher risk of children to develop eczema during childhood if their mothers are subjected to a variety of stress factors, particularly during pregnancy. These findings therefore suggest the importance for pregnant women to avoid exposure to stressful conditions. This in turn highlights the need for the provision of education for pregnant women on the strategies of stress reduction during their maternity leave, and these women should be encouraged to take part in stress reduction programs that are recommended to be made available in local communities. 

Nevertheless, inconsistencies in the findings among the included studies were evident, potentially due to the heterogeneity in the ethnic origin of study participants and methodology utilized in outcome measurement among the studies. In light of this, further research is required to confirm the aforementioned association, preferably with the use of objective measures in stress level measurement and physician-diagnosed eczema for the ascertainment of eczema development, in order to contribute more data for a more reliable conclusion on the positive correlation between maternal stress and childhood eczema.

## Figures and Tables

**Table 1:** The ratings of the reporting quality of the included studies, based on the twelve-item checklist adopted from the STROBE checklist.

Item No.	Item	Braig et al., 2017	Chang et al., 2016	De Marco et al., 2012	Elbert et al., 2017	El-Heis et al., 2017	Hartwig et al., 2014	Larsen et al., 2014	Letourneau et al., 2017	Sausanthaler et al., 2009	Wang et al., 2016	Wen et al., 2011
1	Study design described	Yes	Yes	Yes	Yes	No	Yes	Yes	Yes	Yes	Yes	No
2	Study setting described	Yes	No	Yes	No	Yes	Yes	No	Yes	Yes	Yes	Yes
3	Eligibility criteria of participants described	Yes	No	No	Yes	No	No	Yes	Yes	No	No	No
4	Outcomes and variables defined	Yes	Yes	Yes	Yes	Partial	Yes	Yes	Yes	Yes	Yes	Yes
5	Sources of measures described	Yes	Yes	Yes	Yes	Yes	Yes	Yes	Yes	Yes	Yes	Yes
6	Attempts in addressing biases described	Yes	Yes	Yes	Yes	Yes	Yes	Yes	Yes	Yes	Yes	Yes
7	Rationale of sample size given	No	No	No	No	No	No	No	No	No	No	No
8	Statistical methods described	Yes	Yes	Yes	Yes	Yes	Yes	Yes	Yes	Yes	Yes	Yes
9	Number of participants at each study stage reported	Yes	No	Yes	Yes	No	No	No	Yes	Yes	Yes	Yes
10	Characteristics of participants reported	Yes	Yes	Yes	Yes	Yes	Yes	Yes	Yes	Yes	Yes	Yes
11	Outcome data reported	No	Yes	Yes	Yes	Yes	Yes	No	Yes	Yes	Yes	Yes
12	Adjusted and/or unadjusted estimates reported	Yes	Yes	Yes	Yes	Yes	Yes	Yes	Yes	Yes	Yes	Yes
	Score (Max. 12)	10	8	10	10	7	9	8	11	10	10	9

**Table 2:** The characteristics of the included studies.

Author/Year/Country	Design	Sample Size	Age of Child When Data Collection Was Performed	Methodology in Assessing Eczema	Stress Factor Studied	Confounding Factors Adjusted for	Major Findings
Braig et al., 2017; Germany [17]	Prospective cohort (longitudinal)	787 mother-child pairs	6 months 12 months 24 months	Self-report	Maternal chronic stress Anxiety at pregnancy Depression at pregnancy	child gender child birth weight gestational age maternal atopy maternal smoking during pregnancy maternal body mass index maternal age	Maternal stress and anxiety have positive associations with risk of child having atopic dermatitis (AD) symptoms at 2 years old, but no such associations were observed if we compare maternal stress and anxiety with diagnosis of AD using strict definitions. Adjusted relative risks (RR) when AD diagnosis was by presence of AD-associated symptoms Use of Inventory of Chronic Stress Screening Scale Adjusted RR: 1.5, 95% CI: 1.0–2.3, *p* = 0.05 Use of Hospital Anxiety and Depression Scale-Anxiety subscale Adjusted RR: 1.4, 95% CI: 1.0–2.0 Use of Hospital Anxiety and Depression Scale-Depression subscale Adjusted RR: 1.1, 95% CI: 0.5–2.1 Use of Pregnancy-related anxiety questionnaire Adjusted RR: 1.5, 95% CI: 0.9–2.4 Use of Hair cortisol concentration (comparison between 90th percentile with lowest quartile) Adjusted RR: 1.2, 95% CI: 0.8–2.0 Adjusted relative risks (RR) when AD diagnosis was strict definition of AD Use of Inventory of Chronic Stress Screening Scale Adjusted RR: 1.1, 95% CI: 0.7–1.9 Use of Hospital Anxiety and Depression Scale-Anxiety subscale Adjusted RR: 1.1, 95% CI: 0.7–1.9 Use of Hospital Anxiety and Depression Scale-Depression subscale Adjusted RR: 0.6, 95% CI: 0.2–2.3 Use of Pregnancy-related anxiety questionnaire Adjusted RR: 1.2, 95% CI: 0.6–2.4 Use of Hair cortisol concentration (comparison between 90th percentile with lowest quartile) Adjusted RR: 1.5, 95% CI: 0.8–2.7
Chang et al., 2016; South Korea [13]	Two prospective cohort studies, including Cohort for Childhood Origin of Asthma and Allergic Diseases (COCOA) and Panel Study on Korean children (PSKC) (longitudinal)	COCOA: 973 mother-child pairs PSKC: 1531 mother-child pairs	6 months 1 year 2 years 3 years 4 years	COCOA: clinical diagnosis PSKC: self-report	Prenatal anxiety Prenatal depression Prenatal distress	Maternal age Maternal history of allergic diseases Education Delivery method Child gender Birth season (in COCOA only)	Both studies showed that prenatal depression/anxiety/distress are positively associated with the risk of child to develop atopic dermatitis. COCOA: Prenatal depression: Adjusted OR: 1.31, 95% CI: 1.02–1.69, *p* < 0.05 Prenatal anxiety Adjusted OR: 1.41, 95% CI: 1.06–1.89, *p* < 0.05 PSKC: Prenatal distress: Adjusted OR: 1.85, 95% CI: 1.06–3.25, *p* < 0.05
de Marco et al., 2012; Italy [23]	Retrospective cohort (cross-sectional)	3854 mother-child pairs	Not specified	Self-report	Stressful life events during pregnancy	child gender child age ethnicity parental education parental smoking parental asthma place of residence traffic level near home exposure to industrial pollution exposure to mold pet ownership birth conditions	Exposure to stressful life events by mothers during pregnancy will increase the odds of the child having eczema Adjusted OR: 1.53, 95% CI: 1.11–2.10
Elbert et al., 2017; Holland [18]	Prospective cohort (longitudinal)	5205 mother-child pairs	10 years	Self-report of physician diagnosis	Anxiety during pregnancy Depression during pregnancy	maternal age education level ethnic origin history of allergies/asthma/eczema parity pet ownership body mass index smoking child gender gestational age birth weight	1-unit rise in measures for anxiety and depression is positively associated with eczema risk in children at 10 year, but only anxiety shows statistical significance for this association Anxiety during pregnancy Adjusted OR: 1.21, 95% CI: 1.05–1.39, *p* < 0.05 Depression during pregnancy Adjusted OR: 1.15, 95% CI: 1.02–1.29, *p* > 0.05
El-Heis et al., 2017; United Kingdom [19]	Prospective cohort (longitudinal)	3008 mother-child pairs	6 months 12 months	self-report	Perceived stress in life that affected subjects’ health at preconception Perceived stress in daily living at preconception Psychological distress at preconception	maternal age at birth education smoking in pregnancy parity and eczema child gender gestational age at birth season of birth breastfeeding duration	At 6 months: All stress factors investigated have positive association with infant having eczema, but association is not significant. Perceived stress in life affecting subjects’ health Unadjusted OR: 1.10, 95% CI: 0.98–1.24, *p* = 0.093 Adjusted OR: 1.08, 95% CI: 0.96–1.23, *p* = 0.22 Perceived stress in daily living Unadjusted OR: 1.13, 95% CI: 1.01–1.28, *p* = 0.039 Adjusted OR: 1.12, 95% CI: 0.99–1.28, *p* = 0.072 Psychological distress Unadjusted OR: 1.21, 95% CI: 0.85–1.73, *p* = 0.30 Adjusted OR: 1.24, 95% CI: 0.84–1.83, *p* = 0.28 At 12 months: All stress factors investigated have positive association with infant having eczema, with most having association being significant. Perceived stress in life affecting subjects’ health Unadjusted OR: 1.21, 95% CI: 1.08–1.35, *p* = 0.001 Adjusted OR: 1.21, 95% CI: 1.01–1.37, *p* = 0.002 Perceived stress in daily living Unadjusted OR: 1.16, 95% CI: 1.03–1.30, *p* = 0.014 Adjusted OR: 1.14, 95% CI: 1.00–1.29, *p* = 0.046 Psychological distress Unadjusted OR: 1.43, 95% CI: 1.00–2.04, *p* = 0.044 Adjusted OR: 1.37, 95% CI: 0.93–2.01, *p* = 0.11
Hartwig et al., 2014; Australia [21]	Prospective cohort (longitudinal)	1587 mother-child pairs	6 years 14 years	self-report of physician diagnosis coupled with self-report	Prenatal adverse life events	child gender preterm delivery low birth weight multiple births parity maternal age prenatal and postnatal smoking exposure to dust, paint and air pollution use of antibiotics and medication breastfeeding maternal education maternal history of eczema pet ownership postnatal adverse life events	Increased number of adverse life events experienced by mothers during gestation would result in increased odds of the child having eczema at both 6 years and 14 years of age. The odds increase even further if the adverse life events happen late during the gestational stage. At 6 years: Adverse life events in the first 18 weeks of gestation (comparing between 3 or more life events and no life events): Adjusted OR: 1.41, 95% CI: 0.61–3.29, *p *= 0.75 Adverse life events between 18–34 weeks of gestation (comparing between 3 or more life events and no life events): Adjusted OR: 2.38, 95% CI: 0.63–2.19, *p* = 0.21 At 14 years: Adverse life events in the first 18 weeks of gestation (comparing between 3 or more life events and no life events): Adjusted OR: 1.18, 95% CI: 0.54–2.60, *p* = 0.61 Adverse life events between 18-34 weeks of gestation (comparing between 3 or more life events and no life events): Adjusted OR: 4.19, 95% CI: 1.97–8.89, *p* < 0.01
Larsen et al., 2014; Denmark [22]	Prospective cohort (longitudinal)	32,271 mother-child pairs	18 months 7 years	self-report	Job strain during pregnancy	maternal age parity body mass index smoking alcohol intake gestational age at data collection furry animal ownership maternal atopic disposition use of pain-killers and antibiotics intake of folic acid gender	The level of job strain experienced by mothers during pregnancy increases the risk of their children having atopic dermatitis at 7 years of age. By comparing subjects in high-strain group and those in low-strain group: Adjusted OR: 1.15, 95% CI: 1.02–1.31, *p* < 0.001
Letourneau et al., 2017; Canada [16]	Retrospective cohort (secondary data analysis) (cross-sectional)	242 mother-child pairs	18 months	self-report of physician diagnosis	Prenatal anxiety	maternal sensitivity postnatal depression social support and anxiety maternal asthma	Pregnancy-specific anxiety has a positive impact on the odds of the child having atopic dermatitis at 18 months. Unadjusted OR: 1.57, 95% CI: 0.76–3.27, *p* > 0.05 Adjusted OR: 2.74, 95% CI: 1.04–7.19, *p* < 0.05
Sausenthaler et al., 2009; Germany [20]	Retrospective cohort (secondary data analysis) (longitudinal)	3004 mother-child pairs	0.5 year 1 year 1.5 years 2 years 4 years 6 years	self-report of physician diagnosis coupled with self-report	Maternal stress during pregnancy	study center maternal education maternal age at delivery family history of atopy	Maternal stress during pregnancy is positively correlated with the odds of the child having eczema up to 2 years old only At 1 year old Adjusted OR: 1.24, 95% CI: 0.72–2.13 At 2 years old Adjusted OR: 1.48, 95% CI: 0.95–2.30 At 3 years old Adjusted OR: 1.06, 95% CI: 0.66–1.70 At 4 years old Adjusted OR: 1.06, 95% CI: 0.67–1.68 At 5 years old Adjusted OR: 1.21, 95% CI: 0.76–1.91 At 6 years old Adjusted OR: 1.13, 95% CI: 0.71–1.79
Wang et al., 2016; Taiwan [14]	Prospective cohort (longitudinal)	18,024 mother-child pairs	6 months 3 years	self-report of physician diagnosis coupled with self-report	Postpartum depression	child gender birth weight family history of atopy maternal education prenatal stress breastfeeding family income number of siblings help for children care residence location maternal mental health index	Postpartum depression shows positive association on the risk of questionnaire-diagnosed atopic dermatitis and physician-diagnosed atopic dermatitis among children at 3 years old. Questionnaire-diagnosed atopic dermatitis (through observation of symptoms) Adjusted OR: 1.17, 95% CI: 0.97–1.41, *p* = 0.094 Physician-diagnosed atopic dermatitis Adjusted OR: 1.42, 95% CI: 1.21–1.66, *p* < 0.001
Wen et al., 2011; Taiwan [15]	Prospective cohort (longitudinal)	730 mother-child pairs	6 months 2 years	self-report of physician diagnosis coupled with self-report	Maternal stress during pregnancy	child gender maternal education parental history of atopic diseases	Maternal stress during pregnancy is positively correlated with the odds of the child being diagnosed with atopic dermatitis by a physician at 2 years old. Comparing between high and low reported level of maternal stress during pregnancy: Adjusted OR: 2.3, 95% CI: 1.1–5.3, *p* = 0.036

RR: relative risk; CI: confidence interval; COCOA: Cohort for Childhood Origin of Asthma and Allergic Diseases; OR: odds ratios; PSKC: Panel Study on Korean Children.
